# The Impact of Nutrient Limitation and Harvest Method on the Wet Preservation of *Chlorella vulgaris* Biomass

**DOI:** 10.3390/bioengineering10050600

**Published:** 2023-05-17

**Authors:** Joran Verspreet, Christina M. Kuchendorf, Bärbel Ackermann, Leen Bastiaens

**Affiliations:** 1 Flemish Institute for Technological Research (VITO), Boeretang 200, 2400 Mol, Belgium; leen.bastiaens@vito.be; 2 Institute of Bio- and Geosciences, IBG-2, Forschungszentrum Jülich GmbH, Wilhelm-Johnen-Str., 52428 Jülich, Germany; c.kuchendorf@fz-juelich.de (C.M.K.);; 3 Stadt Erftstadt, Stabsstelle Klimaschutz, Holzdamm 10, 50374 Erftstadt, Germany

**Keywords:** *Chlorella vulgaris*, preservation, nutrient depletion, harvest, organic acids, lipids

## Abstract

The temporary storage of wet algae concentrates enables the decoupling in time of algae harvests and their biorefinery. However, the impact of cultivation and of the harvest conditions on algae quality during preservation is largely unknown. This study aimed to determine the impact of nutrient limitation and of harvest methods on the preservation of *Chlorella vulgaris* biomass. Algae were either well-fed until harvest or received no nutrients for one week, and were harvested by either batch or continuous centrifugation. The organic acid formation, lipid levels, and lipolysis were monitored. Nutrient limitation had a large impact and resulted in lower pH values (4.9 ± 0.4), high levels of lactic acid and acetic acid, and a slightly higher degree of lipid hydrolysis. Concentrates of well-fed algae had a higher pH (7.4 ± 0.2) and another pattern of fermentation products with mainly acetic acid, succinic acid, and, to a smaller extent, lactic acid and propionic acid. The effect of the harvest method was smaller, with, most often, higher lactic acid and acetic acid levels for algae harvested by continuous centrifugation than for those obtained by batch centrifugation. In conclusion, nutrient limitation, a well-known method to enhance algae lipid levels, can impact several quality attributes of algae during their wet storage.

## 1. Introduction

There is growing interest in the commercial use of microalgae for a variety of applications, including food, feed, nutraceuticals, and cosmetics [1,2]. The worldwide production of microalgae has tripled in the last 5 years [2] and is expected to continue expanding significantly in the coming years [3]. *Chlorella* sp. are among the most popular species in terms of cultivation, both on a European [1] and a global level [4]. *Chlorella* sp. biomass has been granted generally regarded as safe (GRAS) status [5] and several *Chlorella* sp. are authorized as food in the EU [6]. *Chlorella* is hence an important group of microalgae from a commercial point of view.

Most algae-related research focuses on algae growth and algae processing. However, the preservation of algae, which is needed to bridge the period between algae cultivation and processing, has received much less attention. This is, however, an inevitable step in the value chain. Indeed, the moment at which algae are ready for harvest depends on the weather conditions and is therefore highly variable. In practice, it is difficult to plan processing immediately after algae cultivation and harvesting. Temporary storage is thus often unavoidable. Wet storage seems to be an attractive approach for short-term preservation because it avoids drying costs. Nevertheless, microbial stability is a concern, and, in some algal species, lipid degradation may also be a risk during wet storage [7]. Cell integrity is known to affect lipid stability during the preservation of *Nannochloropsis oculata* and T-*Isochrysis lutea* [8]. It was suggested that harvesting, and centrifugation in particular, triggers cell disruption and thereby induces lipolysis in T-*Isochrysis lutea* [9]. This finding raises the question of whether algae deterioration can be limited by adjustments in the harvesting method. It can be hypothesized that the impact of harvesting is species-dependent because the cell (wall) structure also varies widely across algae species. It is hence particularly interesting to study the lipid stability–harvest method relation for widely cultivated and commercially interesting algae such as *Chlorella*.

Another knowledge gap in the field of wet algae preservation is the effect of nutrient starvation on algae preservability. Algae cultivation is often ended by a nutrient depletion period to improve lipid content [10]. However, these stressful conditions might adversely affect post-harvest storage and ultimately the final quality of the algae. To date, this topic has received scant attention in the research literature.

The aim of this paper is to study the effect of the harvest method and of nutrient limitation on algae quality during the wet preservation of *Chlorella* concentrates. This should make an important contribution to the optimization of algae storage.

## 2. Materials and Methods

### 2.1. Chlorella Biomass Cultivation, Harvest, and Storage

*Chlorella vulgaris* CCALA (C1) was cultivated in a strictly phototrophic way in V-bags and phytobag modules (total volume 700 l) at an outdoor facility at Forschungszentrum Jülich GmbH (FZJ). The non-axenic outdoor cultures were not heated/cooled, as the general microclimatic conditions were mild (see Supplementary Table S1). Cultures were monitored microscopically and regularly via cell counting (Coulter Counter, Multisizer 3, Beckman, Indianapolis, IN 46268, USA) for a rough overview of the bacteria/debris load and accompanying species or grazers. Microscopy analysis confirmed that *Chlorella* was by far the main algae in the culture. Cultures were supplied with additional CO_2_ (bubbling air enriched to ~1%). More information on algae cultivation is given by Schreiber et al. [11]. Growth was monitored via optical density, and nutrient availability was controlled for total nitrogen and orthophosphate. Nutrients were replenished bi-weekly as necessary with urea and KH_2_PO_4_ as the main N source and P source, respectively. Incident light was determined as global irradiation and PAR outdoors, and temperature as the hourly average from the FZJ meteorological station (50°54′36″ N, 6°24′34″ E, 93 MSL). The relevant growth period lasted from 2nd September to 1st October 2019, with harvests on 9th, 16th, 23rd, and 30th September (regular weekly intervals). The outdoor cultures reached, in the well-fed phase (up to 23rd September), an OD750 of ~7, where an OD750 of 1 equaled roughly ~0.4 g DW/l. For the following week, the culture was divided into a replenished (well-fed) and starving (not fed) culture. The well-fed culture and the culture without a nutrient supply in the last week are further referred to as the well-fed and temporarily unfed cultures, respectively. With deteriorating weather (low light exposure), both culture regimes resulted in OD between 1 and 2.

Both algae cultures (well-fed and temporarily unfed) were harvested by either continuous centrifugation (9000 rpm, feed 1.3 m3/h, Clara 20, Alfa Laval, Lund, Sweden) or by overnight sedimentation (4 °C) and batch centrifugation (10 min at 10,000× *g*, Sorvall LYNX 6000, Thermo Scientific, Waltham, MA, USA) in the laboratory. In the latter case, algae experienced fewer shear forces than during continuous centrifugation. As a result, four different algae concentrates were obtained, and these were stored for 14 days. Algae suspensions (15 mL) were stored in the dark at 8 °C with continuous shaking (orbital movement 150 rpm) either for 7 or 14 days. Next, samples were aliquoted and stored at −20 °C prior to chemical analysis. Samples for lipid analysis were freeze-dried before lipid extraction (Section 2.2), while the other analyses (Section 2.3 and Section 2.4) were performed without a preceding freeze-drying step.

### 2.2. Lipid Analysis

The total lipid content was determined by the chloroform:methanol extraction of freeze-dried algae samples as described by Ryckebosch et al. [12]. The free fatty acid (FFA) level was evaluated by the derivatization of FFA to fatty acid diethylamides as detailed by Kangani et al. [13] and subsequent gas chromatography analysis.

### 2.3. Ethanol Analysis

Ethanol levels were analyzed by high-performance liquid chromatography using an Agilent Hi-Plex H column (300 × 7.7 mm, particle size 8 µm) connected to a refractive index detector. After injection (20 µL), organic acids and ethanol were separated by isocratic elution with 0.01 M H_2_SO_4_ (0.8 mL/min), where the column temperature was kept at 60 °C.

### 2.4. Organic Acid Analysis

Organic acids (lactic acid, acetic acid, succinic acid, propionic acid, and citric acid) were analyzed by ultra-high-performance liquid chromatography–mass spectrometry. Diluted algae concentrates were centrifuged (10 min, 10,000× *g*) and the supernatant was filtered (0.2 µm) and injected (0.2 to 5 µL) on an Atlantis PREMIER BEH AX, 1.7 µm, 2.1 × 100 mm column (Waters Corporation, Milford, MA, USA). The column was kept at 30 °C and eluted (0.350 mL/min) with an aqueous 0.9% formic acid solution for 3 min, aqueous 0.9% formic acid with 10 mM ammonium formate for 5 min, 80% acetonitrile with 0.9% formic acid and 20% of a 0.9% formic acid solution with 50 mM ammonium formate for 1 min, and 3 min with an aqueous 0.9% formic acid solution. For detection, electrospray ionization (ESI) was used with a cone voltage of 15 V, probe temperature at 600 °C, capillary voltage at 0.8 kV, and a desolvation gas flow rate of 1000 l/h. Selected ion recording was performed after ESI ionization in positive mode for acetic acid (m/z 61.0) and propionic acid (m/z 75.0) and in negative mode for lactic acid (89.0 m/z), succinic acid (m/z 117.0), and citric acid (m/z 191.0).

### 2.5. Statistics

Statistical analyses were carried out using Statistica version 12 (Dell Inc., Tulsa, OK, USA, 2015). FFA analyses were performed at least in duplicate, and all other analyses in triplicate. In the storage test, the impact of the (i) nutrient status, (ii) harvest method, and (iii) storage time were studied using a full-factorial design. Factorial analysis of variance (ANOVA) was used to evaluate the effects of these 3 factors on algae quality and to identify interaction effects. The threshold significance level was set at 5%. In case of a positive omnibus test, a posthoc Tukey multiple-comparison test was used to compare the different levels within one factor. To explore the large set of data on the measured algae attributes (organic acid levels, pH, lipid levels, and FFA levels), a principal component analysis (PCA) was performed on the respective average values. For PCA calculations, data were standardized by centering about the mean and scaling by the standard deviation.

## 3. Results

### 3.1. Algae Growth and Harvest

A *Chlorella* culture was either well-fed until harvest or received no nutrients during the last week before harvest to study the impact of nutrient limitation. Growth profiles (Supplementary Figure S1) and culture medium pH values (Supplementary Table S1) were similar for both cultures during the 4-week growth period. Algae were harvested by either batch centrifugation or continuous centrifugation to evaluate the impact of the harvest method. Algae concentrates had a dry matter concentration ranging between 7 and 9% at the start of the preservation test (Supplementary Table S2).

### 3.2. Short-Chain Fatty Acid and pH Levels

**Figure 1:**
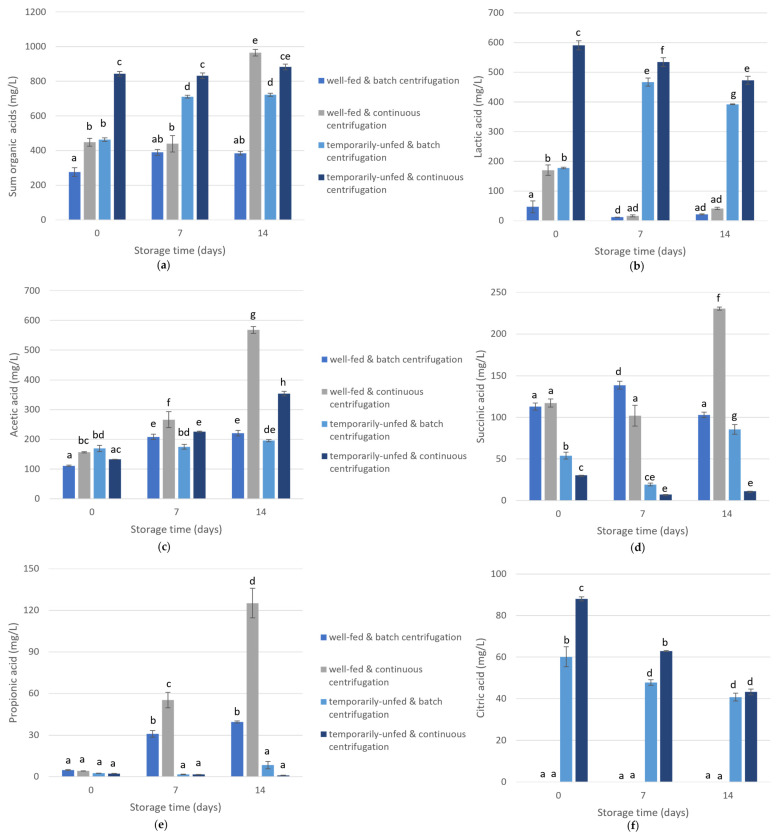
Short-chain fatty acid concentrations during algae storage. ANOVA analysis results can be found in Supplementary Information (Supplementary Tables S3–S8). Values within one figure that are not labeled by the same lowercase letter are significantly different. Error bars indicate standard deviations on triplicate measurements. A small correction was made to account for differences in the organic matter content at t_0_ (Supplementary Table S2); organic matter concentrations were divided by the corresponding t_0_ organic matter concentration and multiplied by the average t_0_ organic matter concentration.

The total short-chain fatty acid levels were significantly affected by all studied variables and most of their interactions (Supplementary Table S3). The harvest method (F = 350, *p* < 0.001) and culture status (F = 325, *p* < 0.001) had the strongest impact. In general, higher short-chain fatty acid levels were detected after continuous centrifugation than after batch centrifugation, and higher levels were also observed in the temporarily unfed culture than in the well-fed culture (Figure 1a). Lactic acid and acetic acid were the main metabolites in the temporarily unfed culture, while acetic acid and succinic acid were the main metabolites in the well-fed culture (Figure 1).

Lactic acid levels (Figure 1b) were mainly affected by the culture nutrient status at harvest (F = 10,300, *p* < 0.001) and, to a smaller extent, by the harvest method (F = 117, *p* < 0.001), time (F = 15, *p* < 0.001), and their interactions (Supplementary Table S4). Lactic acid concentrations were higher in the temporarily unfed cultures, especially after harvest by continuous centrifugation. Acetic acid (Figure 1c) was predominantly affected by the storage time (F = 937, *p* < 0.001), harvest method (F = 829, *p* < 0.001), and their interaction (F = 426, *p* < 0.001), although other variables had an impact as well (Supplementary Table S5). Succinic acid levels were mainly affected by the culture nutritional status at harvest (F = 3283, *p* < 0.001), with higher levels for the well-fed culture (Figure 1d), and were less influenced by the other factors (Supplementary Table S6). Propionic acid concentrations became high only for the well-fed culture after storage (Figure 1e, Supplementary Table S7). Citric acid (Figure 1f) was only detected in the temporarily unfed algae. Ethanol levels are not shown because most values were below the limit of quantification (LOQ, 48.6 mg/L). Ethanol was only present in the temporarily unfed cultures at t_0_ (above the detection limit (24.3 mg/L) but below LOQ) after continuous centrifugation and 7 days of storage (56.5 ± 3.9 mg/L), and after batch centrifugation and 7 days of storage (53.5 ± 5.8 mg/L) and 14 days of storage (50 ± 2.7 mg/L).

**Figure 2:**
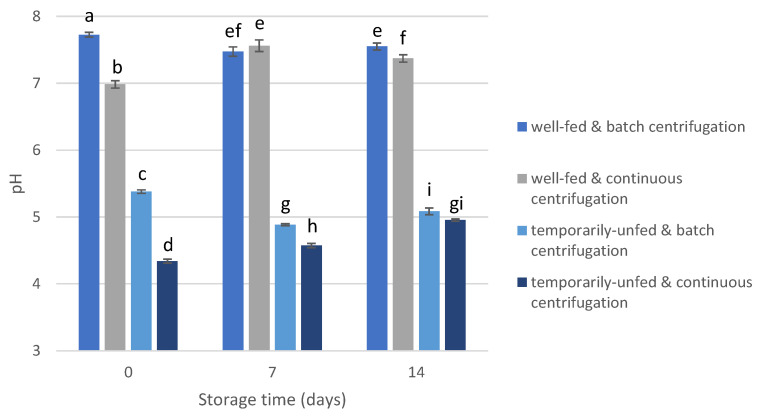
pH values of algae concentrates during storage. Values that are not labeled by the same lowercase letter are significantly different.

The pH of the algae concentrates (Figure 2) was mainly affected by the culture status at harvest time (F = 25,435, *p* < 0.001, Supplementary Table S9), with clearly lower pH values for temporarily unfed algae concentrates (4.87 ± 0.35) than for well-fed concentrates (7.44 ± 0.24). The effect of the centrifugation method (F = 570, *p* < 0.001), storage time (F = 27, *p* < 0.001), and the interaction effects (Supplementary Table S9) was smaller.

### 3.3. Lipid Analysis

Total lipid content was not significantly affected by any of the tested storage factors (*p* > 0.05, Supplementary Table S10), with average lipid levels ranging between 21.4% and 24.8% of the dry matter (not shown).

**Figure 3:**
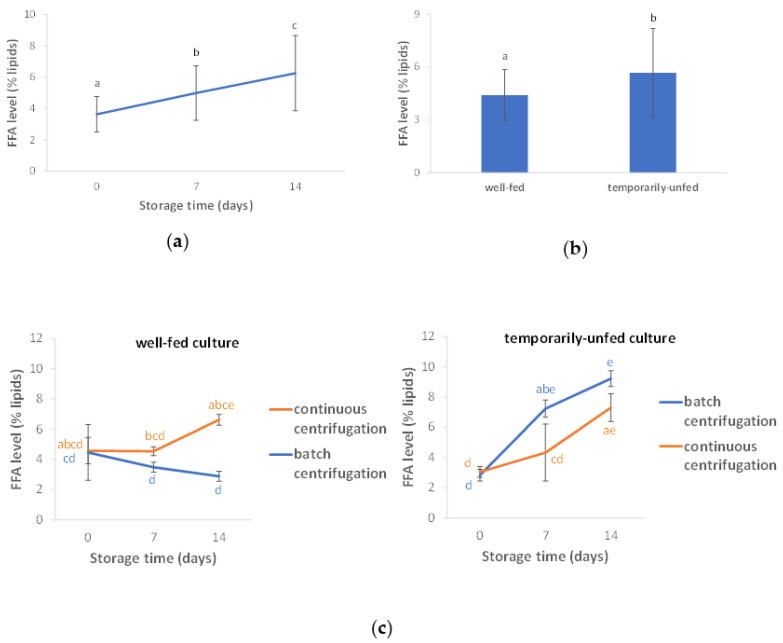
(**a**) Free fatty acid (FFA) levels as a function of storage time, (**b**) FFA levels as a function of culture status at harvest, and (**c**) FFA levels broken down across all studied factors. Error bars indicate standard deviations. FFA levels within Figure 3a that are not labeled by the same lowercase letter are significantly different. The same holds for FFA levels in Figure 3b and for FFA levels within Figure 3c.

The free fatty acid (FFA) levels were affected by the storage time (F = 30, *p* < 0.001), culture status at harvest (F = 17, *p* = 0.001), and all possible interaction effects (Supplementary Table S11). FFA levels increased with increasing storage time (Figure 3a). Moreover, FFA concentrations were higher for the temporarily unfed algae than for algae that were well-fed until harvest (Figure 3b), although the concentration difference was limited. The harvest method (continuous centrifugation versus batch centrifugation) had no significant impact (*p* = 0.872). However, there was an interaction effect between the storage time, culture status at harvest, and harvest method (*p* = 0.003, Figure 3c). After storage of the well-fed culture, FFA levels were lower after batch centrifugation than after continuous centrifugation, but this was not seen for the nutrient-depleted culture.

### 3.4. Correlation Analysis

**Figure 4:**
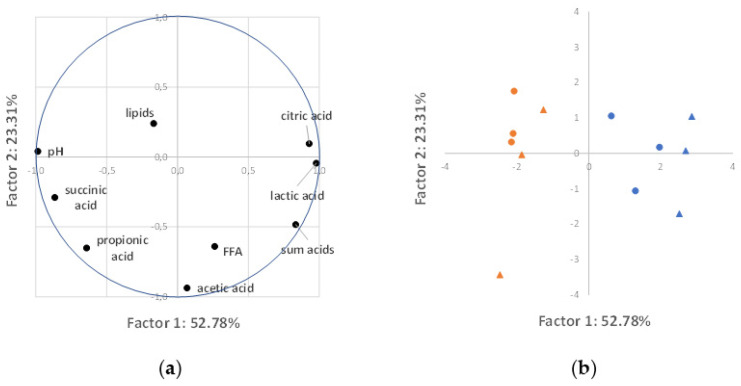
PCA results. (**a**) Projection of the variables on the factor plane. (**b**) Projection of the average observations on the factor plane. Orange and blue marks in Figure 4b represent observations from the well-fed and temporarily unfed algae, respectively. Triangles and circles in Figure 4b represent data from continuous and batch-centrifuge-harvested algae, respectively.

To explore the entire dataset of algae attributes (organic acid levels, pH, lipid levels, and FFA levels), a PCA was performed. PCA simplifies the analysis of a high-dimensional dataset by transforming the data into fewer dimensions, the so-called principal components, where most of the variation in the data can be described [14]. The cumulative proportion of variance accounted for by the two first principal components was 76.1%, with the first and second factors accounting for 52.8% and 23.3% of the variance, respectively (Supplementary Table S12). The pH, lactic acid, citric acid, succinic acid, and total organic acid level were the main contributors of the first factor, while acetic acid was the main contributor to the second factor (Figure 4a and Supplementary Table S13). Projecting the data based on the first two principal components (Figure 4b) shows that results from well-fed algae concentrates (orange markers) and those from temporarily unfed algae concentrates (blue markers) form two distinct clusters along the horizontal axis of the first principal component.

**Figure 5:**
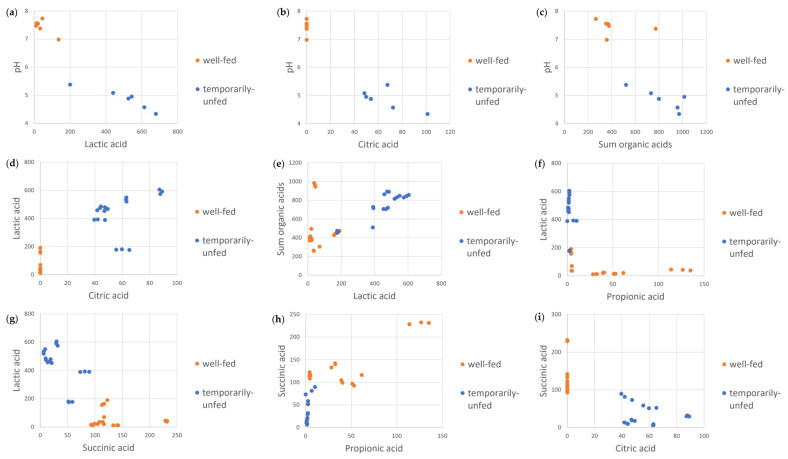
(**a**) Scatterplots of the average pH and lactic acid levels, (**b**) average pH and citric acid levels, and (**c**) average pH and sum of all organic acid levels. (**d**–**i**) show scatterplots for individual measurements of organic acid levels (5d: lactic acid vs citric acid. 5e: sum of all organic acids vs lactic acid. 5f: lactic acid vs propionic acid. 5g: lactic acid vs succinic acid, 5h: succinic acid vs propionic acid, 5i: succinic acid vs citric acid). Data from well-fed and temporarily unfed algae are represented by orange and blue markers, respectively.

The PCA analysis also suggests that there are several strong correlations between the variables. The Spearman’s rank correlation coefficient (ρ) between average values was calculated to quantify the degree of correlation (Supplementary Table S14). For the variables that correlated the most (i.e., the nine highest ρ coefficients), scatterplots are shown in Figure 5. The pH value correlated negatively with the lactic acid levels (ρ = −0.90, Figure 5a), citric acid levels (ρ = −0.89, Figure 5b), and the sum of all measured organic acid levels (ρ = −0.88, Figure 5c). Lactic acid levels correlated positively with citric acid levels (ρ = 0.88, Figure 5d) and with the sum of all measured organic acid levels (ρ = 0.77, Figure 5e) and correlated negatively with propionic acid levels (ρ = −0.83, Figure 5f) and with succinic acid levels (ρ = −0.80, Figure 5g). Succinic acid levels correlated positively with propionic acid levels (ρ = 0.83, Figure 5h) and negatively with citric acid levels (ρ = −0.82, Figure 5i). There is again a clustering of the results of well-fed algae concentrates (orange markers) and those of temporarily unfed algae concentrates (blue markers).

## 4. Discussion

**Figure 6:**
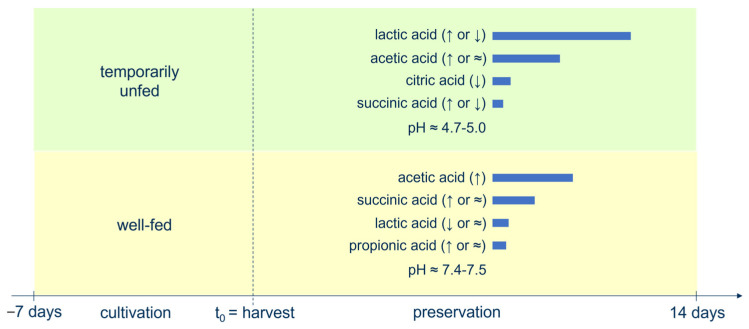
Visual summary of the impact of the algae culture’s nutritional status on organic acid and pH levels during storage. The length of the blue bars is proportional to the organic acid concentrations averaged over the day 0–day 14 preservation period. The symbols between brackets (↓, ↑ or ≈) indicate the evolution of organic acid concentrations during storage.

The aim of this work was to evaluate the impact of the nutritional status at harvest time (temporarily unfed vs. well-fed until harvest) and of the harvest method (continuous vs. batch centrifugation) on the preservation of *Chlorella vulgaris* biomass. The culture’s nutritional status at harvest had clearly the largest impact on the studied algae parameters. Its impacts on organic acid and pH levels are visualized in Figure 6.

### 4.1. The Impact of Nutritional Status at Harvest

The well-fed and temporarily unfed algae formed two distinct clusters in the PCA analysis. The most obvious difference was seen for the pH values (4.87 ± 0.35 for temporarily unfed algae and 7.44 ± 0.24 for well-fed algae). Interestingly, the well-fed and temporarily unfed algae cultures had still similar pH values a few hours before harvest (7.2 and 7.6, respectively), while there was a clear pH difference at the start of the storage test (i.e., a few hours after harvest). Apparently, only the combination of nutrient limitation and algae harvest induced a strong pH decrease. One can speculate that the algae, once weakened by the nutrient-deficient regime, were more prone to cell lysis during harvest. This could result in the higher release of cell content and cell debris, increasing the availability of substrates for aerobic degradation (as long as O_2_ is available) and fermentative degradation (once all O_2_ is consumed). The latter process can result in the production of high organic acid levels and a pH decrease. Accordingly, pH values correlated with total organic acid levels (ρ = −0.88) and lactic acid levels (ρ = −0.90), and high lactic acid levels were noted for the temporarily unfed algae concentrates only. Lactic acid is usually not a major product of green algal fermentation [15] but can be formed by several types of bacteria, especially lactic acid bacteria. These lactic acid bacteria, particularly *Lactobacillus* bacteria, thrive in acidic, carbohydrate-rich environments [16] and can outcompete with other bacteria because they are more resistant to low pH values. The medium pH in turn can steer the type of fermentation products that are being formed in anoxic environments by bacteria [15] and algae [17]. Many bacteria produce mainly acetate, ethanol, formate, and low levels of succinate at a neutral pH, while producing lactate instead of acetate and formate in more acidic environments [15]. The type of organic acids being formed by bacteria also depends on the available substrates. Ultimately, in the absence of O_2_, most bacteria reduce partially oxidized metabolic intermediates, forming mainly lactate, succinate, and ethanol, and excrete these metabolites together with formate and acetate [15]. Moreover, for algae, the fermentation pattern depends largely on the algae species and on environmental conditions such as the available carbon source. Typical fermentation products under anoxic conditions for green algae are acetate, ethanol, formate, glycerol, lactate, H_2_, and CO_2_ in the case of starch fermentation [15].

It is also possible that the one-week no-feeding regime began to form the bacterial community even before harvesting and therefore the algal–bacterial consortia responded differently at harvest. The nitrate concentration, for instance, was suggested to be a primary driver in the bacterial community composition of outdoor *Nannochloropsis* sp. cultures [18]. Finally, several studies indicated that nutrient depletion may also increase the algal production of extracellular polymeric substances (EPS), another possible fermentation substrate. However, literature has emerged that offers contradictory findings, and the effect of nutrient depletion on EPS appears not yet fully understood [19].

Taken together, the differences between temporarily unfed algae and well-fed algae may be caused by different degrees of cell lysis during harvest and/or differences in the composition of the bacterial community or by diverging EPS secretion. All these factors can steer the interplay between the algal–bacterial consortium, fermentation metabolism, and pH. Accordingly, temporarily unfed and well-fed algae formed two distinct clusters in the PCA analysis (Figure 4b), with the pH as the main contributor to the first PCA factor (Figure 4a) and correlating strongly with lactic acid (ρ = −0.90), citric acid (ρ = −0.89), total organic acid (ρ = −0.88), and succinic acid (ρ = 0.76) levels. The temporarily unfed cluster was associated with high levels of acetic and mainly lactic acid, and lower levels of citric and succinic acid. The well-fed cluster, on the other hand, was associated with the presence of acetic acid, succinic acid, and, from 7 days of storage onwards, propionic acid.

The observed differences between temporarily unfed and well-fed algae will have a major influence on several algae quality attributes. Firstly, the low pH values will limit the growth of many microorganisms, including many pathogens and most food spoilage organisms [16]. This is obviously a beneficial effect when food or feed applications are envisioned. Secondly, the reduced growth of most bacteria reduces the associated dry matter losses. Because of this, the conditions observed here (low pH and high lactic acid levels) are exactly the conditions targeted during ensilage, which enables the long-term storage (e.g., 180 days) of algae [20]. Thirdly, low pH values might inhibit the bacterial formation of malodorous metabolites such as propionic acid and butyric acid. These two organic acids have a strong off-odor and have been shown to contribute to the bad smell of algae stored under inappropriate conditions [21]. Wendt et al. (2017) observed that adding organic acids or inorganic acids reduced butyrate levels during the anaerobic incubation of *Scenedesmus obliquus*. This was explained by the inhibition of butyrate-producing species by the low pH. In another study, acetic acid addition (50 mM) prevented the increase in propionic and butyric acid during the wet storage of *Nannochloropsis* concentrates. In the current study, low pH values correlated with the absence of propionic acid seen in nutrient-depleted algae (butyric acid was not measured).

Although nutrient deprivation is a well-known method to stimulate lipid accumulation [10], nutrient limitation had no impact on the total lipid levels in this study. Possibly, algae were under-active during the week of nutrient deficiency because of the poorer weather conditions. Differences might become clearer when the nutrient supply is stopped in conjunction with better weather conditions. The level of FFA, a measure for lipid hydrolysis, was significantly affected by the nutrient status at harvest, and also by the storage time. As expected [8,9,21], FFA levels generally increased with increasing storage time. Balduyck et al. hypothesized that this is triggered by a loss of cell integrity induced at harvest, resulting in increased contact between lipids and lipases [8]. As suggested above, the nutrient limitation could have weakened the algae, and so enforced cell lysis during harvest. Accordingly, temporarily unfed algae generally had higher FFA levels than well-fed algae. However, differences were small in the current study, and all FFA levels were rather low. Differences might become clearer when a less robust algae species is used.

### 4.2. The Impact of Harvest Method

PCA analysis revealed no clear clustering according to the harvest method (Figure 4b) but, for some individual parameters, the harvest method had a significant impact according to the factorial ANOVA analysis. The most obvious effect was seen on lactic acid and acetic acid levels, but also the pH and total organic acid levels were affected. In general, higher levels of lactic acid, acetic acid, and total organic acids were detected after continuous centrifugation than after batch centrifugation. Again, these differences might be related to the different degrees of fermentation and different levels of fermentation substrates available, because of the different degrees of cell lysis. Indeed, one can expect that more cell rupture takes place during continuous centrifugation as cells experience higher shear forces than during batch centrifugation. However, this explanation is not consistent with the lack of effect of the harvest method on FFA levels (*p* = 0.87). It is noteworthy that not only the intracellular material released by cell lysis can feed fermentation processes, but also extracellular polymeric substances (EPS). Some EPS are closely associated with algae cells [19] and their removal efficiency may depend on the used harvest method. It is also possible that a higher degree of cell lysis is needed to trigger FFA formation in robust algae cells, while organic acid formation occurs more easily. In line with this, the storage of *Nannochloropsis* concentrates in a previous study triggered no FFA increase in one test, while organic acid levels (acetic acid and propionic acid) increased under the same storage conditions [21]. In any case, the lack of effect seen here with C. vulgaris, which is considered to have a rigid cell wall [22], does not exclude the possibility that gentle harvesting protects the cells of more sensitive algae, such as Porosira glacialis [23] or T-Isochrysis [8], against cell lysis and lipolysis. Batch centrifugation as applied here is, however, difficult to scale up and to perform in a commercial production environment. Membrane filtration then becomes a more attractive harvest method.

## 5. Conclusions

Nutrient limitation had a major impact on the parameters monitored in this study. Although there was no impact on lipid levels, nutrient limitation clearly reduced the pH and favored the formation of lactic acid and, to a minor extent, of citric acid. In fact, it created an environment similar to that of ensilage storage. The differences in algae harvested by batch and continuous centrifugation were smaller, and mainly limited to differences in lactic acid, acetic acid, and total organic acid levels and pH. In this regard, the nutrient status should be considered when designing the algal growth and processing chain. Nutrient limitation can lower the pH, which in turn can have positive effects such as reducing the risk of pathogen growth and bacterial off-odor formation. However, a note of caution is due here since algae had low light exposure in the last growth week and only one algae species was tested in this study. Algae growth conditions can be expected to have an important impact, as can the algae species.

## Data Availability

Not applicable.

## Supplementary Materials

The following supporting information can be downloaded at: https://www.mdpi.com/article/10.3390/bioengineering10050600/s1, Figure S1: Well-fed algae culture growth curve, Figure S2: The temporarily unfed algae culture growth curve; Table S1: Outdoor temperature during algae growth and culture pH values, Table S2: Dry matter and organic matter concentrations of algae at the start of the preservation test, Table S3: Results of factorial ANOVA analysis. Impact on total organic acid levels, Table S4: Results of factorial ANOVA analysis. Impact on lactic acid, Table S5: Results of factorial ANOVA analysis. Impact on acetic acid, Table S6: Results of factorial ANOVA analysis. Impact on succinic acid, Table S7: Results of factorial ANOVA analysis. Impact on propionic acid, Table S8: Results of factorial ANOVA analysis. Impact on citric acid, Table S9: Results of factorial ANOVA analysis. Impact on pH, Table S10: Results of factorial ANOVA analysis. Impact on lipid content, Table S11: Results of factorial ANOVA analysis. Impact on FFA levels, Table S12: PCA eigenvalues and percentage of variance associated with each eigenvalue, Table S13: Factor–variable correlations (factor loadings), based on correlations, Table S14: Spearman rank order correlations among average values.
