# Supplementary material for: The Impact of Nutrient Limitation and Harvest Method on the Wet Preservation of Chlorella vulgaris Biomass
Source: Bioengineering (Basel). 2023 May 17;10(5):600. doi: 10.3390/bioengineering10050600 (PMC10215344; doi:10.3390/bioengineering10050600)
Supplement: Supplementary file 1 [file bioengineering-10-00600-s001.zip › bioengineering-2398059-supplementary.pdf]

The impact of nutrient limitation and harvest method on the wet preservation of *Chlorella vulgaris* biomass

Joran Verspreet[1], Christina M. Kuchendorf[2], Bärbel Ackermann[2,*1], and Leen Bastiaens[1]

Supplementary information

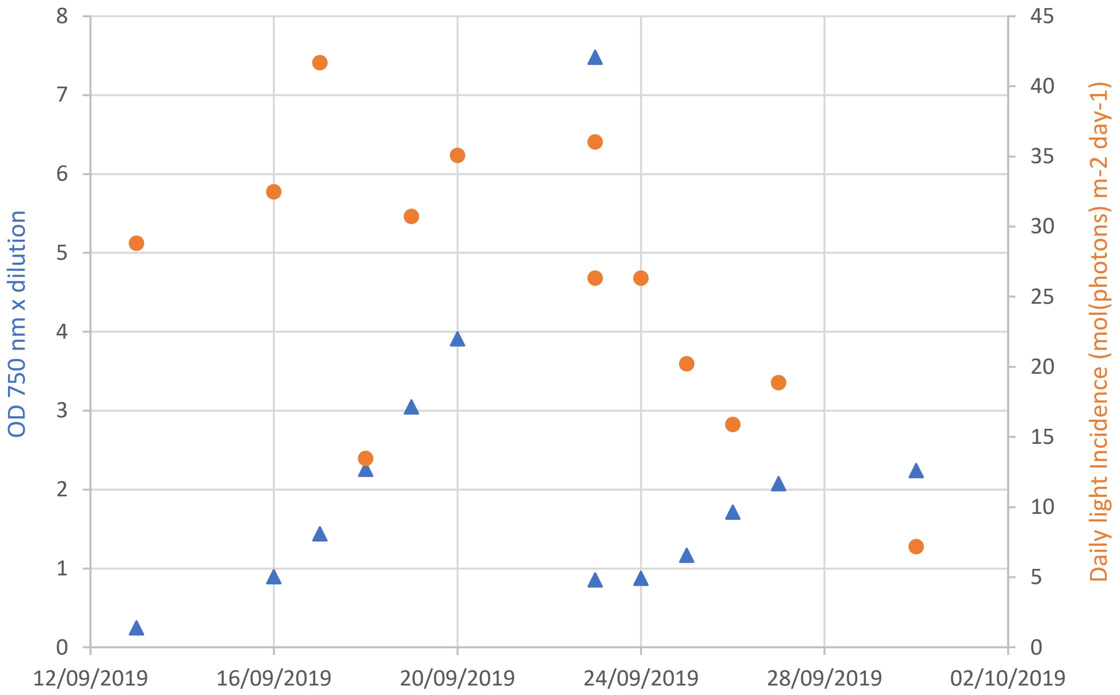

Supplementary Figure S1. Well-fed algae culture growth curve. The (OD at 750 nm x dilution rate) is plotted by blue triangles. Daily light incidence is shown by orange dots.

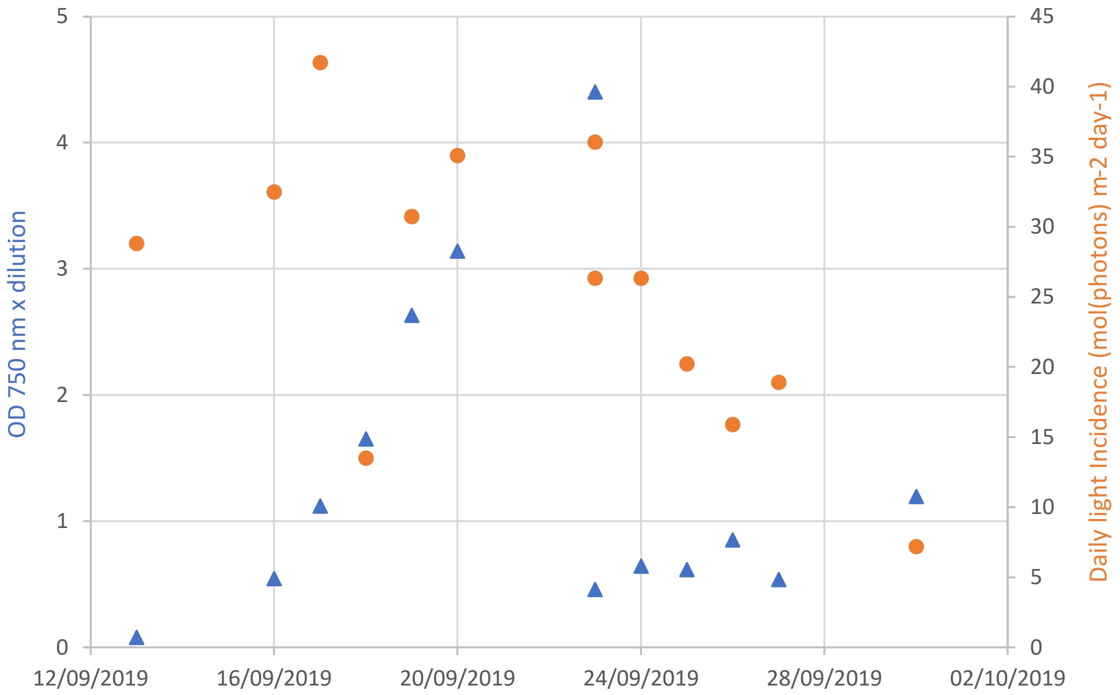

Supplementary Figure S2. The temporarily-unfed algae culture growth curve. The culture received no nutrients in the last week. The (OD at 750 nm x dilution rate) is plotted by blue triangles. Daily light incidence is shown by orange dots.

Supplementary Table S1. Outdoor temperature during algae growth and culture pH values.

	**Maximum temperature (°C) day N-1**	**Minimum temperature (°C) day N-1**	**Temperature range (°C) day N-1**	**Average temperature (°C) day N-1**	**pH well-fed culture**	**pH nutrient-depleted culture**
13/09/2019	18	9.7	8.3	13.1	no data	no data
16/09/2019	20.8	13.7	7.1	17.5	7.23	7.69
17/09/2019	20.4	12.3	8.1	15.3	7.14	7.68
18/09/2019	18.1	7.9	10.2	13.9	6.96	7.34
19/09/2019	17.2	4.9	12.3	11.2	6.9	7
20/09/2019	17.1	4.4	12.7	10.6	6.78	7.27
23/09/2019	19.8	4.1	15.7	12.1	6.57	7.42
23/09/2019	19.8	12.5	7.3	16.0	no data	no data
24/09/2019	19.8	12.5	7.3	16.0	6.92	7.35
25/09/2019	19.7	12.2	7.5	14.8	6.95	7.46
26/09/2019	18.5	13	5.5	15.8	6.86	7.33
27/09/2019	19	14.5	4.5	16.8	8.01	8.17
30/09/2019	20	12.1	7.9	15.8	7.18	7.56

Supplementary Table S2. Dry matter and organic matter concentrations of algae at the start of the preservation test.

Nutritional status at harvest	Harvest method	DM (%)	OM (%)
Well-fed	Continuous centrifugation	6.7 ± 0.0	6.4 ± 0.1
Well-fed	Batch centrifugation	8.1 ± 0.7	7.7 ± 0.6
Temporarily unfed	Continuous centrifugation	9.2 ± 0.2	9.2 ± 0.2
Temporarily unfed	Batch centrifugation	8.9 ± 0.2	9.0 ± 0.2

Supplementary Table S3. Results factorial ANOVA analysis. Impact on total organic acid levels.

Effect	F	P-value
time	82	<0.001
harvest method	350	<0.001
culture status at harvest	325	<0.001
time*harvest method	46	<0.001
time*culture status at harvest	34	<0.001
harvest method*culture status at harvest	0.7	0.396
time*harvest method*culture status at harvest	38	<0.001

Supplementary Table S4. Results factorial ANOVA analysis. Impact on lactic acid.

Effect	F	P-value
time	15	<0.001
harvest method	957	<0.001
culture status at harvest	10300	<0.001
time*harvest method	385	<0.001
time*culture status at harvest	257	<0.001
harvest method*culture status at harvest	325	<0.001
time*harvest method*culture status at harvest	98	<0.001

Supplementary Table S5. Results factorial ANOVA analysis. Impact on acetic acid.

Effect	F	P-value
time	937	<0.001
harvest method	829	<0.001
culture status at harvest	165	<0.001
time*harvest method	427	<0.001
time*culture status at harvest	118	<0.001
harvest method*culture status at harvest	165	<0.001
time*harvest method*culture status at harvest	52	<0.001

Supplementary Table S6. Results factorial ANOVA analysis. Impact on succinic acid.

Effect	F	P-value
time	181	<0.001
harvest method	1	0.324
culture status at harvest	3283	<0.001
time*harvest method	82	<0.001
time*culture status at harvest	65	<0.001
harvest method*culture status at harvest	367	<0.001
time*harvest method*culture status at harvest	368	<0.001

Supplementary Table S7. Results factorial ANOVA analysis. Impact on propionic acid.

Effect	F	P-value
time	337	<0.001
harvest method	185	<0.001
culture status at harvest	1052	<0.001
time*harvest method	85	<0.001
time*culture status at harvest	301	<0.001
harvest method*culture status at harvest	244	<0.001
time*harvest method*culture status at harvest	121	<0.001

Supplementary Table S8. Results factorial ANOVA analysis. Impact on citric acid.

Effect	F	P-value
time	198	<0.001
harvest method	128	<0.001
culture status at harvest	8101	<0.001
time*harvest method	39	<0.001
time*culture status at harvest	198	<0.001
harvest method*culture status at harvest	128	<0.001
time*harvest method*culture status at harvest	39	<0.001

Supplementary Table S9. Results factorial ANOVA analysis. Impact on pH.

Effect	F	P-value
time	27	<0.001
harvest method	570	<0.001
culture status at harvest	25435	<0.001
time*harvest method	246	<0.001
time*culture status at harvest	44	<0.001
harvest method*culture status at harvest	45	<0.001
time*harvest method*culture status at harvest	17	<0.001

Supplementary Table S10. Results factorial ANOVA analysis. Impact on lipid content.

Effect	F	P-value
time	0.25	0.781
harvest method	0.085	0.774
culture status at harvest	0.243	0.628
time*harvest method	0.11	0.896
time*culture status at harvest	0.983	0.393
harvest method*culture status at harvest	0.192	0.667
time*harvest method*culture status at harvest	0.553	0.585

Supplementary Table S11. Results factorial ANOVA analysis. Impact on FFA levels.

Effect	F	P-value
time	29.66	<0.001
harvest method	0.03	0.872
culture status at harvest	16.93	0.001
time*harvest method	3.32	0.059
time*culture status at harvest	24.72	<0.001
harvest method*culture status at harvest	28.38	<0.001
time*harvest method*culture status at harvest	8.03	0.003

Supplementary Table S12. PCA eigenvalues and percentage of variance associated with each eigenvalue.

Eigenvalue number	Eigenvalue	% Total variance	Cumulative % total variance
1	4.8	52.8	52.8
2	2.1	23.3	76.1
3	1.0	11.0	87.1
4	0.7	8.1	95.2
5	0.3	3.2	98.3
6	0.1	0.9	99.3
7	0.1	0.6	99.9
8	0.0	0.1	100.0
9	0.0	0.0	100.0

Supplementary Table S13. Factor-variable correlations (factor loadings), based on correlations.

	Factor 1	Factor 2	Factor 3	Factor 4	Factor 5	Factor 6	Factor 7	Factor 8	Factor 9
acetic acid	0.07	-0.94	-0.03	-0.20	0.25	0.13	0.05	0.00	-0.01
propionic acid	-0.64	-0.65	0.09	-0.32	-0.12	-0.21	0.02	-0.01	0.00
lactic acid	0.98	-0.04	0.14	0.01	-0.03	-0.04	-0.13	0.01	-0.01
succinic acid	-0.87	-0.29	0.10	-0.02	-0.36	0.15	-0.06	0.00	0.00
citric acid	0.93	0.10	0.01	-0.24	-0.23	0.02	0.12	0.05	0.00
sum acids	0.83	-0.48	0.17	-0.18	0.00	0.01	-0.10	0.01	0.02
pH	-0.99	0.04	0.01	0.03	0.13	-0.02	-0.06	0.07	0.00
lipids	-0.17	0.24	0.95	-0.03	0.07	0.02	0.04	0.00	0.00
FFA	0.26	-0.64	0.12	0.71	-0.07	-0.04	0.04	0.01	0.00

*Supplementary Table S14. Spearman Rank Order Correlations among average values. *Marked correlations are significant at P <0.05.*

Variable	acetic acid	propionic acid	lactic acid	succinic acid	citric acid	sum	pH	Lipids	FFA
acetic acid	1.00	0.16	0.01	-0.13	0.01	0.47	-0.15	-0.41	0.41
propionic acid	0.16	1.00	-0.83*	0.83*	-0.76*	-0.62*	0.75*	-0.07	-0.12
lactic acid	0.01	-0.83*	1.00	-0.80*	0.88*	0.77*	-0.90*	0.09	0.17
succinic acid	-0.13	0.83*	-0.80*	1.00	-0.82*	-0.63*	0.76*	0.26	-0.09
citric acid	0.01	-0.76*	0.88*	-0.82*	1.00	0.76*	-0.89*	-0.04	-0.13
sum	0.47	-0.62*	0.77*	-0.63*	0.76*	1.00	-0.88*	-0.02	0.22
pH	-0.15	0.75*	-0.90*	0.76*	-0.89*	-0.88*	1.00	0.01	-0.15
Lipids	-0.41	-0.07	0.09	0.26	-0.04	-0.02	0.01	1.00	-0.13
FFA	0.41	-0.12	0.17	-0.09	-0.13	0.22	-0.15	-0.13	1.00
